# Overview of common oral lesions

**DOI:** 10.51866/rv.37

**Published:** 2022-08-01

**Authors:** Esha Zahid, Osama Bhatti, Muhammad Abdullah Zahid, Michael Stubbs

**Affiliations:** 1 BHSc, MDent, LaTrobe University, Melbourne, Australia Email: esha.zahid23@gmail.com; 2 MBBS, FRACGP, Monash Health, Melbourne, Australia; 3 MBBS, Emergency Department, Casey, Hospital, Monash Health, Melbourne, Australia; 4 BDS, MDS, FRACDS, MRACDS, Epworth Freemasons Hospital, Melbourne, Australia

**Keywords:** Oral medicine, Oral lesions, Primary health care, Dentistry

## Abstract

This article summarises common oral lesions that clinicians may face in everyday practice by categorising them by clinical presentation: ulcerated lesions, white or mixed white—red lesions, lumps and bumps, and pigmented lesions. The pathologies covered include recurrent aphthous stomatitis, herpes simplex virus, oral squamous cell carcinoma, geographic tongue, oral candidosis, oral lichen planus, pre-malignant disorders, pyogenic granuloma, mucocele and squamous cell papilloma, oral melanoma, hairy tongue and amalgam tattoo. The objective of this review is to improve clinician knowledge and confidence in assessing and managing common oral lesions presenting in the primary care setting.

## Introduction

Clinicians encounter various oral lesions in everyday practice. Oral lesions can arise from a range of different aetiologies: infective, idiopathic, inflammatory, reactive and neoplastic changes. A clinician must obtain a thorough clinical history and have adequate knowledge of the signs and symptoms, such as the location of the oral mucosal lesion and its size, colour and morphology to make a proper diagnosis.

This article aims to review common oral lesions that practitioners are faced with in everyday practice and provide an overview of the following conditions: recurrent aphthous stomatitis, herpes simplex virus, oral squamous cell carcinoma, geographic tongue, oral candidosis, oral lichen planus, pre-malignant disorders, pyogenic granuloma, mucocele and squamous cell papilloma, oral melanoma, hairy tongue and amalgam tattoo. The oral acording to their typical clinical presentations, such as ulcerated lesions, white or mixed white—red lesions, lumps and bumps, and pigmented lesions.

## Ulcerated Lesions

### Recurrent Aphthous Stomatitis (RAS)

RAS is the most common ulcerative disease and is present in approximately 20% of the general population. However, the prevalence varies from 5% to 50% depending on the population group, and in some cases, personal and work-related stress.^1^ In children, the incidence of RAS is heavily influenced by the presence of RAS in parents.^1^ Children with parents positive for RAS have a 90% chance of developing the condition, compared to 20% in children whose parents do not have RAS. RAS appears to peak in the second decade of life and becomes less frequent in the later years of life.^1^

#### Risk Factors

The exact aetiology of RAS is unknown, but several factors have been suggested as possible causes.^1^

#### Local, microbial, nutritional and other factors

Trauma is a causative agent for RAS.^1^ RAS patients often have ulcerations at trauma sites due to toothbrushing, dental treatment or biting of the oral mucosa.^2^ Dysregulated salivary composition, xerostomia and stresses have also been linked with an increase in RAS.^1^

It is a common misconception to confuse RAS with microbial infections, but several studies have established that HSV, Helicobacter pylori and EBV do not cause RAS.^1^

Low levels of iron, zinc, folate, and vitamins B1, B2, B6 and B12 have been found twice as commonly in RAS patients, and up to 20% of individuals with RAS may have a nutritional deficiency.^2^

Other factors including high levels of stress and hypersensitivity to certain foods such as chocolate and peanuts have been implicated in some patients with RAS.^2^ However, with regards to tobacco, the incidence of RAS was lower in smokers compared to non-smokers. This may be because tobacco tends to increase keratinisation of the oral mucosa, hence rendering the mucosa less susceptible to oral ulceration.^2^

#### Underlying medical disorders

Systemic disorders associated with RAS are nutritional deficiencies including anaemia, Behcet’s syndrome, HIV, cyclic neutropenia and MAGIC syndrome, to name a few. RAS-like ulceration is a clinical feature of Behcet’s syn drome and may involve major and herpetiform ulcers.^1,2^

Individuals with HIV may also have RAS, which is seen in approximately 5%–15% of HIV-positive patients.^2^ RAS occurs more frequently in immunocompromised patients than others.^2^

Cyclic neutropenia involves a decrease in circulating neutrophils. The distinguishing features of this disease include RAS, mastoiditis, otitis and febrile episodes in infancy.^2^

#### Clinical Features

The three main presentations of RAS are described in **Table 1**^3^

**Table 1:** Clinical features of minor, major and herpetiform RAS. Adapted from Montgomery-Cranny J, Wallace A, Rogers H, Hughes S, Hegarty A, Zaitoun H. Management of recurrent aphthous stomatitis in children. *Oral Medicine Dental Update.* 2015;42(6):564–572.

	MINOR RAS	MAJOR RAS	HERPETIFORM RAS
**Morphology**	Round or oval lesions, erythematous halo, grey-white pseudomembrane^3^ (see *Figure 1*)	Round or oval lesions, erythematous halo, grey-white pseudomembrane^3^ (see *Figure 2*)	Small, deep multiple ulcers, coalesce into one, irregular margins^3^
**Distribution**	Buccal and labial mucosa, lips, tongue, floor of mouth^3^	Lips, soft palate, pharynx, gingiva^3^	Buccal and labial mucosa, tongue, floor of mouth, gingiva^3^
**Number of ulcers**	1-5	1-10	10-100
**Size**	<10 mm	>10 mm	2-3 mm
**Gender predilection**	Equal	Equal	Female
**Incidence**	Patients aged 5-19 80% with RAS^3^	10% with RAS^3^	Less than 10% with RAS^3^
**Healing period**	10-14 days, no scarring^3^	>6 weeks, high risk of scarring^3^	<4 weeks, scarring uncommon^3^
**Treatment**	Topical corticosteroids; tetracycline mouth rinse	Topical, systemic, intralesional corticosteroids; immunosuppressants	Topical, systemic corticosteroids; tetracycline mouth rinse

**Figure 1:**
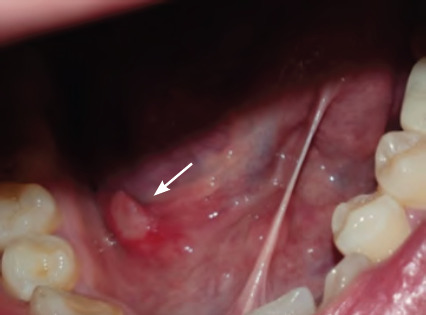
Minor recurrent aphthous ulcer on right floor of mouth

**Figure 2:**
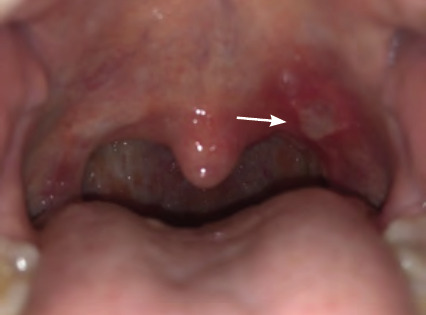
Major recurrent aphthous ulcer on the palate

#### Treatment

Drug therapy is usually considered for individuals who present with severe pain and difficulty eating or who experience multiple episodes of RAS each month.^2^ Protective emollients such as Zilactin or Orabase, and topical agents and anaesthetics such as lidocaine or benzocaine, can be used to palliate the pain from minor ulcers.^1,2^ Tetracycline mouth rinses and NSAIDs can reduce the pain and healing time.^1^ Topical corticosteroid therapy is effective in most cases, but systemic corticosteroids such as prednisone may be required in severe cases of major RAS.^1,2^ Referral to an oral medicine specialist may be required in severe cases of RAS.

### Herpes Simplex Virus Infections (HSV)

Herpes simplex virus types 1 and 2 are highly prevalent worldwide, with both types 1 and 2 able to infect multiple sites of the body, including the oral cavity. Data from the United States showed that 40% of those under 20 years of age had positive antibodies against HSV-1, and after the age of 70, that number rose to 65%.^5^

#### Clinical Features

**Figure 3:**
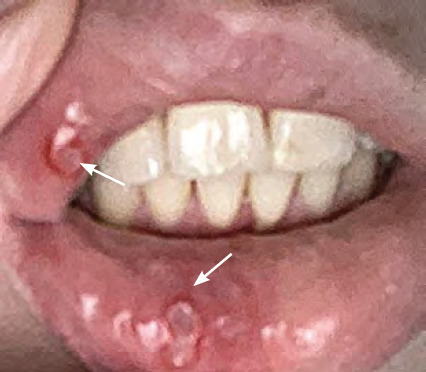
Primary herpetic stomatitis — lesions on upper and lower lip

**Figure 4:**
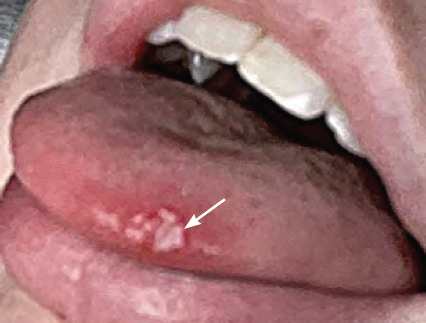
Primary herpetic stomatitis - lesion on tongue

**Figure 5:**
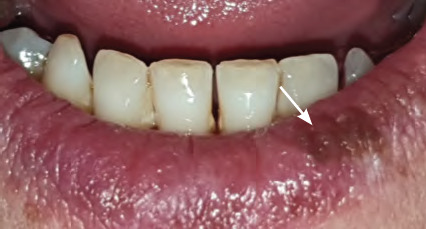
Herpes labialis of the lower left lip (crusted lesion)

HSV infections manifest as primary herpetic stomatitis and secondary (recurrent) herpes labialis. Primary infections often occur during childhood, when children may contract the virus asymptomatically or present with a viral prodrome of fever, headache and cervical lymphadenopathy, followed by an eruption of vesicular lesions within the gingiva (primary herpetic gingivostomatitis). These vesicular lesions may appear on the gingiva, borders of the lips or perioral skin region as solitary lesions or clusters (**Figures 3** and **4**). The vesicles are prone to rupture and may also appear crusted or as sores. Primary episodes often self-resolve within 10 to 14 days, after which the virus lies dormant in the trigeminal nerve ganglion^7^. Secondary herpes labialis is recurrent herpes that presents as a localised crop of vesicles at the vermillion border, as seen in **Figure 5**. Oral HSV shedding into saliva occurs in an estimated 10% of humans. Certain triggers, such as UV light trauma, stress, fatigue or menstruation, may reactivate the virus at later stages, leading to shorter episodic outbreaks.^7^

#### Treatment

Herpetic mouth lesions are diagnosed clinically but can be confirmed by taking a polymerase chain reaction swab. Because episodes self-resolve, most mild cases can be treated supportively with adequate fluid intake and analgesic antipyretics such as paracetamol. Systemic antivirals such as valaciclovir or famciclovir are useful in neonates, pregnant women and immunocompromised patients, especially when used early in the disease state.^8^

Recurrent episodic outbreaks from reactivation of the dormant virus result in milder presentations called ‘cold sores’. Patients are most infectious within the first 24 hours of lesions appearing, and although most are mild and will self-resolve, larger lesions can be treated with topical acyclovir cream or famciclovir.^8^ For patients who experience severe recurrences or are immunocompromised with chronic lesions, a role exists for daily prophylactic antivirals.^8^

### Oral Squamous Cell Carcinoma

Oral squamous cell carcinoma is the most common oral malignancy, accounting for more than 90% of oral cancers.^9^ Oral squamous cell carcinoma tends to affect adults older than 40 years of age and roughly twice as many males as females.^10^ Although oral squamous cell carcinoma can occur anywhere in the oral cavity, it is most often found on the lateral borders of the tongue, followed by the gingiva and alveolar mucosa, the floor of the mouth and the ventral surface of the tongue. In southeastern Asian countries where areca nut— and tobacco-chewing are common, oral squamous cell carcinoma is commonly found on the buccal mucosa.^9^

#### Risk Factors

Risk factors for oral squamous cell carcinoma include alcohol use, tobacco use, betel quid— and areca nut—chewing, and HPV infection.^9^ Tobacco can release pro-carcinogens that can lead to cancer development with a dose—risk relationship.^11^ Alcohol is another risk factor for the development of oral cancer and produces cytotoxic agents when metabolised, in addition to nutritional deficiencies, immune deficiencies and local effects on mucosal linings.^11^

Additional risk factors for the development of oral squamous cell carcinoma include solar radiation, immunosuppression (e.g., transplant patients, HIV/ AIDS) and HPV infection, particularly HPV 16 and 18.^12^ Solar radiation most commonly involves damage to the lower lip because it receives more direct sun exposure.^9^

#### Clinical Features

Early-stage oral squamous cell carcinoma is often painless and asymptomatic, resulting in a delay in diagnosis. More than half of oral carcinoma cases are advanced at the time of diagnosis.^9^

**Figure 6:**
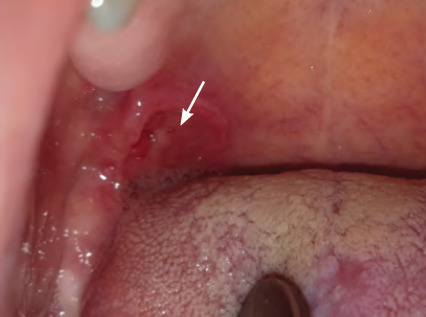
Squamous cell carcinoma of the right pterygomandibular region

**Figure 7:**
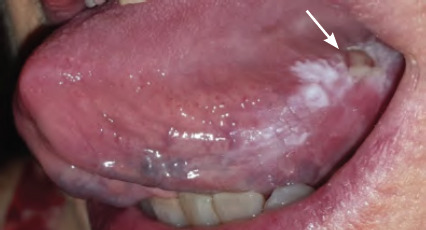
Squamous cell carcinoma of the left ventral surface of the tongue

Oral squamous cell carcinomas can vary in presentation, from non-healing ulcers in the oral cavity, lumps or swellings to dental symptoms such as ill-fitting prostheses^10^ (**Figures 6** and **7**). Additional features may include erythroplakia, leukoplakia, pain or numbness, ulcers with fissures or raised exophytic margins, and non-healing extraction sockets.^12^ Two common malignant features to observe are induration (increased tissue density) and fixation (lack of tissue mobility).^13^ Any suspicious lesion in the oral cavity that does not resolve after 3 weeks should be investigated further and referred promptly to a specialist.^10^

#### Treatment

The management of oral squamous cell carcinoma mainly involves surgical excision, with the approach directed by tumour characteristics such as site, location, size, depth and bone involvement.^12^ Metastatic cases of oral squamous cell carcinoma involve cervical lymph nodes 80% of the time, and as such, lymph node resection plays an important role in management.^12,14^ Radiotherapy and/or chemotherapy may be used in locally advanced squamous cell carcinoma cases.^14^ Despite advances in medical treatment options, the 5-year survival rate for oral cancer remains around 50%, so emphasis on early detection and management is vital in improving patient outcomes.^15^

## White Or Mixed White-Red Lesions

### Oral Candidosis

Oral candidosis is the overpopulation of commensal yeast, most commonly Candida albicans in the mouth. This is often termed an opportunistic infection because changes in the oral microflora enable the proliferation of the Candida organism.^16^

#### Risk Factors

Common predisposing factors include recent broad-spectrum antibiotic use, dentures, inhaled corticosteroids without rinsing the mouth afterwards, smoking, diabetes and immunosuppression (e.g. AIDS, haematological malignancies, chemotherapy).^16^ Dry mouth, called xerostomia, is also a significant cause of oral candidosis.

#### Clinical Features and Treatment

Oral candidosis may be further divided into four categories: pseudomembranous, erythematous, hyperplastic and denture-induced stomatitis.^17^ **Table 2** outlines the clinical features and treatment regimens for oral candidosis.

The diagnosis of oral candidosis is usually made on the clinical appearance, but an oral swab or smear in many cases is helpful to confirm the diagnosis. First-line treatment consists of topical antifungal agents such as nystatin liquid, amphotericin lozenges or miconazole gel. Refractory cases should be further investigated for an underlying cause. This may also include the use of fluconazole or ketoconazole.^8^

**Table 2:** Types, features and treatment of oral candidosis

Type	Features	Treatment
Pseudomembranous	Most common type; presents with creamy white coating on mucosa that can be wiped away, revealing underlying erythema^17^ (**Figures 8** and **9**)	Topical antifungal therapy^8^
Erythematous (atrophic)	Common with prolonged steroid or antibiotic use; presents as erythematous areas that are sensitive, painful (burning) and smooth (due to depapillation)^17^ (**Figure 10**)	Topical antifungal therapy^8^
Hyperplastic	Often chronic; white patches or plaques that cannot be removed^17^ (**Figure 11** )	May resemble leukoplakia or oral cancer; consider referral to specialist for biopsy^8^
Denture-induced stomatitis	Common in up to 60% of denture wearers; presents with erythematous areas at denture-bearing areas and may be associated with angular cheilitis^16,17^ (**Figure 12**)	Dental review to assess fit of dentures and denture hygiene, followed by topical antifungal therapy to mouth and dentures^16,17^

**Figure 8:**
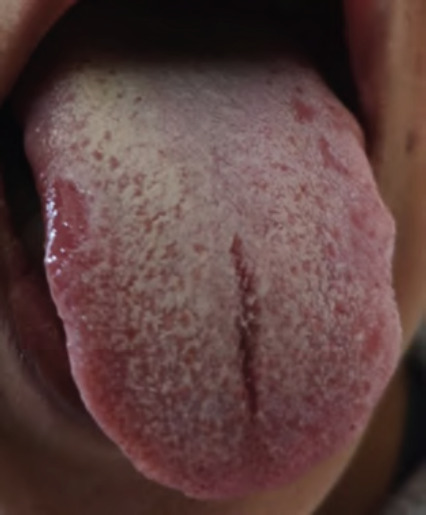
Pseudomembranous candidosis of the tongue

**Figure 9:**
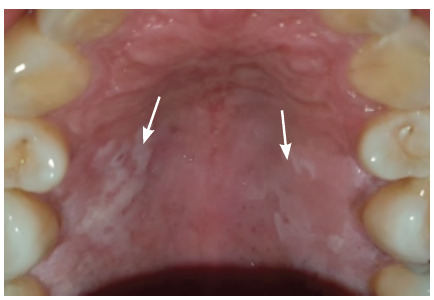
Pseudomembranous candidosis of the hard palate (confirmed by oral swab)

**Figure 10:**
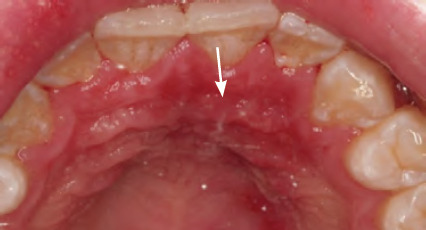
Erythematous candidosis of the hard palate

**Figure 11:**
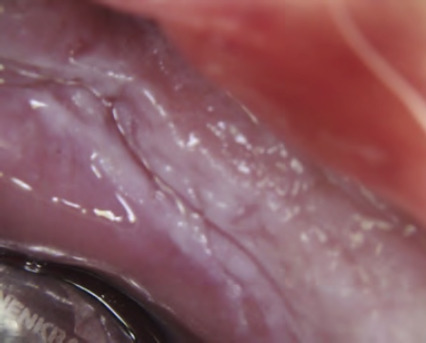
Hyperplastic candidosis associated within the edentulous ridge 47-48-retromolar region in a diabetic with poor denture hygiene

**Figure 12:**
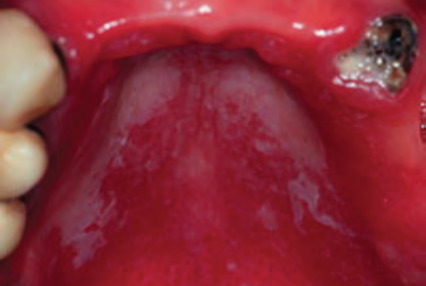
Denture-induced stomatitis

### Oral Lichen Planus (OLP)

OLP is a chronic inflammatory disease that affects the oral mucosa.^18^ It is a subtype of lichen planus and is associated with cell-mediated immunological dysfunction.^18^ OLP has a prevalence of 1–2%, usually affecting adults with a male-to-female ratio of 1:2.^19,20^

Although the exact aetiology of OLP is unknown, it is thought to result from an immune-mediated mechanism involving both CD4+ helper (Th 1) and CD8+ cytotoxic T-cells, causing epithelial damage.^7,20^

#### Clinical Features

The most common sites for OLP are the gingiva, tongue and buccal mucosa, followed by the vermillion border of the lip and the labial mucosa; lesions on the floor of the mouth and the palate are rare.^20,21^ The incidence of lesions occurring on the buccal mucosa is 73%–95.5%, and bilateral involvement can be seen in approximately 82% of patients.^20^

OLP can be classified into six types, which are described in **Table 3**

**Table 3:** Types, features and incidence of OLP

Type	Features	Incidence	Treatment
Reticular	Presents as small, white keratotic papules connected by a white, lacy network known as Wickham’s striae;^33,36^ usually asymptomatic but can cause taste alteration and burning sensation if on the tongue^21^ (**Figures 13** and **14**)	64.8%^20^	Generally, treatment is not required^20,21^
Plaque-like	White, smooth keratotic lesions, sometimes with striations^20^	5.7%^20^	If asymptomatic, no treatment required; if symptomatic, topical corticosteroids can be used^20,21^
Papular	Small keratotic lesions of approximately 1 mm in diameter^20^	2.3%^20^	Topical corticosteroids e.g. dexamethasone, triamcinolone;^20,21^ systemic corticosteroids e.g. prednisolone if topical therapy ineffective^21^
Atrophic/erythematous	Regions of muscle atrophy with thinned epithelium in conjunction with previous reticular lesions^20,21^	4.3%^20^	Same as above
Erosive	Red or erythematous areas with central ulceration of varying degrees and bordered by fine white striae; characterised by lesions, ulcers and sometimes bullae^20,21^ (**Figure 15**)	22.9%^20^	Same as above
Bullous	Severe erosions resulting in rupture of epithelium and bullae formation^20^	Rare^20^	Same as above

Approximately two thirds of individuals affected by OLP experience symptoms. Most symptoms are associated with atrophic and erosive (ulcerative) forms of OLP. These symptoms include mucosal roughness, burning sensation, irritation, xerostomia, bleeding and dysgeusia.^20,21^ If OLP is localised to the gingiva, it can have clinical features of desquamative gingivitis (**Figures 16** and **17**).^21^

OLP has the potential for malignant change into squamous cell carcinoma. It has been classified as a potentially malignant disorder by the World Health Organization. OLP requires regular monitoring for any malignant changes. The reported frequency of malignant change ranges from 0% to 5.8%.^20^

Diagnosis can be made from both clinical findings and biopsy. The authors of this paper recommend a biopsy, both to confirm the diagnosis and to exclude the presence of any dysplastic change.^20^

**Figures 13 and 14:**
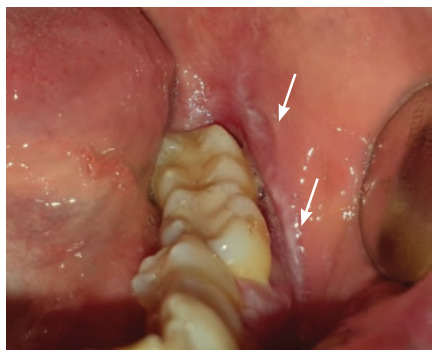

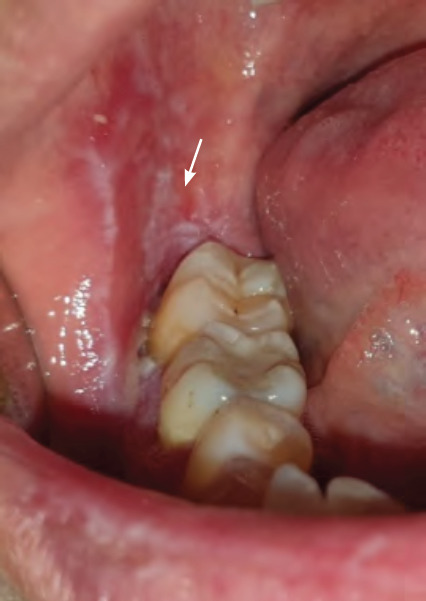
Reticular form of oral lichen planus on buccal mucosa

**Figure 15:**
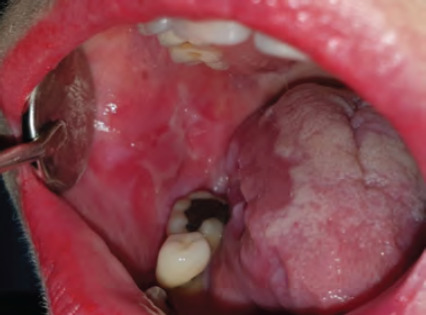
Erosive oral lichen planus

**Figures 16 and 17:**
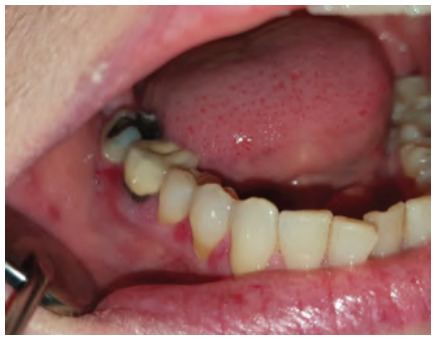

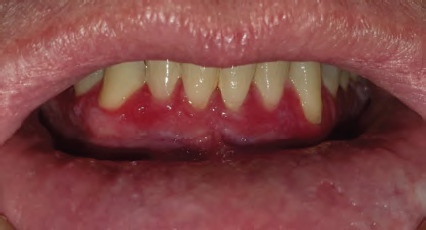
Gingival oral lichen planus areas of desquamation or loss of the epithelial surface appearing intensely red — desquamative gingivitis

#### Treatment

Since the aetiology of OLP is unknown, no cure exists for this disease. The goal of treatment is to relieve the symptoms and reduce inflammation to allow for healing.^20,21^ Symptomatic lesions such as erosive or erythematous OLP require treatment because they are painful.^20,21^ The first line of treatment includes topical corticosteroids such as dexamethasone and triamcinolone.^20,21^

Systemic corticosteroids such as prednisolone are another treatment option, but they are reserved for acute exacerbations of OLP, where topical therapy has been ineffective or where mucosal sites are also affected.^21^

### Geographic Tongue

Geographic tongue is a benign condition with an incidence of 2%–3%. It presents as smooth red areas of depapillation across the dorsum and lateral borders of the tongue.^22^ Geographic tongue is a recurrent condition, and the lesions migrate to different areas of the tongue from time to time.^23^

#### Clinical Features

**Figures 18 and 19:**
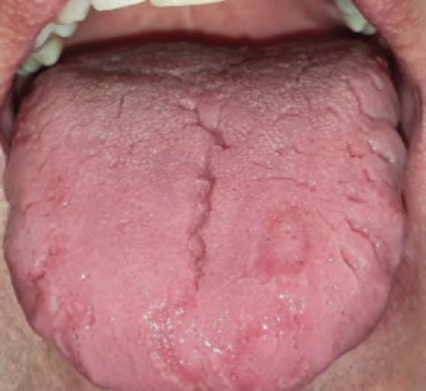

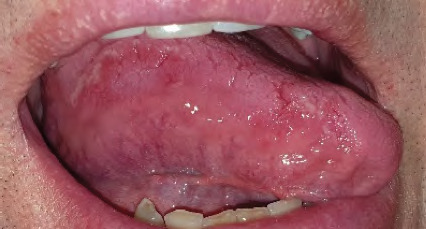
Geographic tongue on dorsal and lateral surfaces of the tongue

Clinical features include an erythematous area surrounded by white margins depicting regeneration of papilla and keratin (**Figures 18** and **19**). The aetiology of geographic tongue is unknown.^22^ It is usually a painless condition, but some individuals may experience a burning sensation and sensitivity to acidic and spicy foods.^23^

#### Treatment

A biopsy is usually not required but can be taken in certain cases to rule out malignant conditions. The lesions of geographic tongue generally resolve spontaneously, and no treatment is needed. Reassuring the patient that lesions are benign and self-limiting is necessary. Any burning sensation can be alleviated through the use of topical steroids and topical anaesthetics.^23^

### Pre-Malignant Disorders

**Figure 20:**
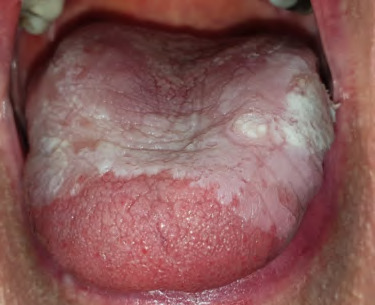
Leukoplakia (biopsied with diagnosis of oral lichen planus and secondary oral candidosis)

**Figure 21:**
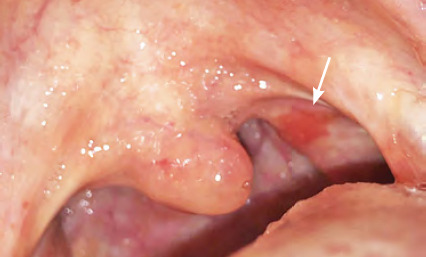
Erythroplakia

Oral potentially malignant disorders are defined as potentially pre-malignant lesions or disorders found in the oral cavity. These disorders include leukoplakia, erythroplakia, oral submucous fibrosis, actinic cheilitis and OLP.^24^ Early identification and treatment of these lesions is recommended to prevent the development of oral squamous cell carcinoma. Oral leukoplakia refers to white plaques of the oral mucosa with unknown cause. Leukoplakia may increase the risk of malignancy and should be differentiated from other benign conditions such as candidiasis, hairy leukoplakia, lupus erythematous and morsication (**Figure 20**).^25^ Smokers are six times more likely to develop leukoplakia, and it carries a 1% annual risk of malignant transformation.^25^ Leukoplakia has two main types - homogenous and non-homogenous. The homogenous type is uniformly white in appearance and is flat and thick, whereas the non-homogenous type appears as a combined red and white lesion, sometimes also called erythroleukoplakia or speckled leukoplakia. It can appear speckled or nodular within any site of the oral cavity.^25^ Due to the very high risk of malignant transformation, these lesions may require surgical excision. Erythroplakia refers to red patches within the oral mucosa that do not clinically appear as any other identifiable condition (**Figure 21**).^25^

**Figure 22:**
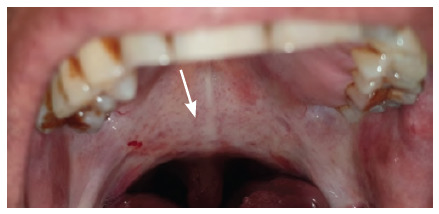
Submucosal fibrosis in a patient who used betel leaf frequently (the pallor of the soft palate is an extensive fibrous change within the submucosa)

Oral submucous fibrosis is a chronic condition of the oral cavity and results in inflammation and fibrosis of the submucosal layer. Patients often present with difficulty opening the jaw, intolerance to spicy food, or a burning sensation with clinical features of mucosal stiffening and fibrosis^25^ (**Figure 22**). Oral submucous fibrosis has an estimated 10-year malignancy transformation risk of 7.6%.^26^

**Figure 23:**
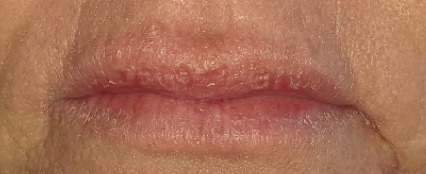
Dry and fissured lips (called cheilitis) secondary to oral candidosis with solar elastosis (actinic cheilitis)

Actinic cheilitis is a condition affecting the lip, caused by exposure to solar UV radiation resulting in histopathological changes such as elastosis, chronic inflammatory infiltrate, vasodilation and hyperkeratosis. It can progress to squamous cell carcinoma.^27^ Clinically, actinic cheilitis presents with lip dryness, atrophy, scaly lesions, ulcerations and loss of vermilion border (**Figure 23**). The incidence of actinic cheilitis is around 31%.^28^ These multifocal changes most often occur on the lower lip of typically fair-skinned individuals above the age of 40 who have a history of significant sun exposure.^27^

A biopsy is required to correctly establish a definitive diagnosis and assess the degree of dysplasia as well as to differentiate potentially premalignant disorders from other inflammatory and atrophic lesions.

## Lumps And Bumps

### Mucocele

**Figure 24:**
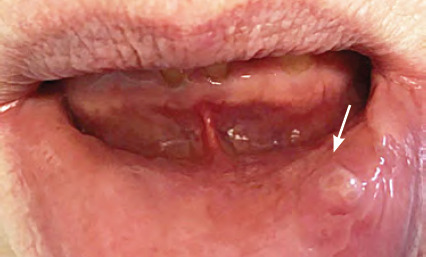
Mucocele on lower lip

Mucoceles are cavities filled with mucus and are benign soft-tissue masses that are usually asymptomatic.^29^ They are mucus-containing cystic lesions of the minor salivary glands and can appear as single or multiple, round, smooth, raised, smooth fluctuant nodules (**Figure 24**).^29^ Mucoceles are classified as a mucous extravasation cyst (MEC) or mucous retention cyst (MRC) depending on the histological features as outlined in **Table 4**. Ranula is a type of mucocele found on the floor of the mouth.^29^ Mucoceles are most prevalent between the ages of 10 and 19 years. Oral mucoceles do not cause significant issues, but individuals may experience discomfort and problems with speech, swallowing and mastication depending on the size of the lesion and the location.^29^ **Table 4** describes the features and treatment of mucoceles.

**Table 4:** Clinical features and treatment of mucoceles

	MEC	MRC	Ranula
**Age Group**	Children and young adults^29^	Older adults^29^	-
**Clinical and Histological Features**	No epithelial lining. Macrophages, eosinophils and plasma cells are present.^30^ Granulation tissue is present.^30^ Dome-shaped, fluctuant nodule with bluish translucency due to spilled mucin. Older lesions are firmer.^29^	True cyst due to presence of epithelial lining. Mucin and inflammatory debris are present.^30^ Similar appearance to MEC but pain present and mucus or pus may be expressed.^29^	Blue dome-shaped fluctuant swelling that can elevate the tongue and is present lateral to the midline.^29^ Larger than other mucoceles, sometimes reaching several centimetres in size.^29^
**Causes**	Rupture of minor salivary gland duct and mucin spilling into surrounding soft tissue.^29^ Mechanical trauma such as cheek or lip biting.^29^	Due to mucous retention in the duct or acini due to a sialolith or calculi obstructing the duct. The narrow ductal opening is blocked, causing swelling and irritation.^29^ Irritation can be due to toothpastes, hydrogen peroxide mouth rinses and anti-plaque solutions.^29^	Arises from leaking of saliva from sublingual gland, Wharton’s duct or ducts of Rivini.^29^
**Site**	Buccal and labial mucosa with a size less than 1.5 cm^29^	Floor of the mouth, upper lip, hard palate, maxillary sinus^29^	Floor of the mouth^29^
**Treatment**	Marsupialisation, dissection, carbon dioxide lasers or complete excision including total removal of feeder gland to minimise recurrence^29^		

### Squamous Cell Papilloma (SCP)

SCP is a benign and asymptomatic exophytic mass presenting in the oral cavity.^31^ It is caused by HPV types 6 and 11. Risk factors include smoking, dietary deficiencies, hormonal changes and other infections.^31,32^ The two types of SCP are isolated-solitary and multiple-recurring. Isolated-solitary are generally found in adults, whereas multiple-recurring are usually found in children.^31^ HPV can transmit via skin-to-skin, oral or sexual contact with an infectious person, as well as the possibility of vertical transmission from mother to child.^31^

#### Clinical Features

**Figure 25:**
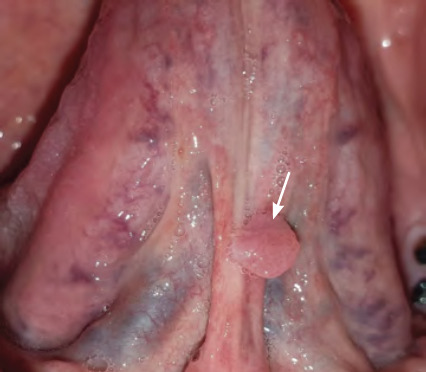
Squamous papilloma of the lingual frenum

SCP is a localised proliferation appearing cauliflower-like and arising from the underlying soft tissue. It appears as a single lesion and grows to reach a maximum of 1 cm.^31,32^ The most common sites include the palate (37.84%), tongue (29.73%), lips and gingiva. It most commonly affects patients between the ages of 30 and 50 years, although it can also be found in paediatric patients (**Figure 25**).^31^,^32^

#### Treatment

Treatment of SCP includes surgical excision of the entire lesion, including some surrounding tissue. Other treatment modalities include laser such as CO2 and ER,Cr:YSGG, electrocautery and cryosurgery.^31,32^ Recurrence is uncommon, except in patients with HIV.^31^

### Pyogenic Granuloma

Pyogenic granuloma is a non-neoplastic rapidly growing vascular lesion that most commonly occurs on the skin or mucous membranes. The aetiology is unclear, but it is believed to result from inflammatory hyperplasia secondary to trauma, chronic irritation, medications or hormone factors such as pregnancy.^33^

#### Clinical Features

**Figure 26:**
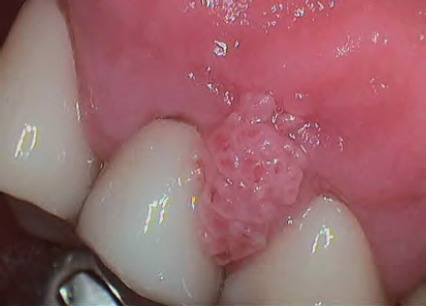
Pyogenic granuloma

Oral pyogenic granuloma presents as a localised red papule that is smooth or lobulated, on a pedunculated or sessile base. The size of lesions can vary from a few millimetres, and a characteristic history of rapid growth is a common clinical feature. They are also haemorrhagic and can bleed easily from minor trauma, and some may ulcerate.^33^ The majority of lesions occur in the gingiva, particularly the maxillary gingiva, but they may also affect the lips, mucosa and tongue (**Figure 26**).^34^

#### Treatment

Excision of pyogenic granulomas, especially during pregnancy, carries a high recurrence rate.^33^ Management of pyogenic granuloma may range from clinical observation for small, asymptomatic lesions to surgical excision.^33^

## Pigmented Lesions

### Oral Melanoma

Primary oral malignant melanomas can be considered rare, with estimates suggesting that less than 1% of melanomas occur on mucosal surfaces.^35^ The incidence of oral melanomas increases with age. The aetiology of oral melanomas is unknown, but some risk factors include pre-existing pigmented naevi, infection, trauma from ill-fitting prostheses and tobacco consumption.^36^

#### Clinical Features

**Figure 27:**
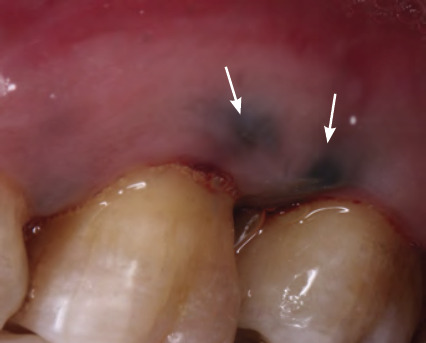
Oral melanoma

Most oral melanomas appear as black, brown, white, grey, purple or red lesions, but a third may appear amelanocytic (**Figure 27**). Additional features may include ulceration, central nodules and satellite lesions.^36^ Oral mucosal melanomas occur most commonly on the hard palate (40%) or gingival surfaces (28%).^37^ Most (85%) are invasive at the time of diagnosis, with a poor 5-year survival rate of around 15%.^37^ Diagnosis is confirmed through a biopsy.^36^

#### Treatment

Treatment options include surgery to excise the tumour as well as radiotherapy, chemotherapy and immunotherapy.^36^

### Amalgam Tattoo

**Figure 28:**
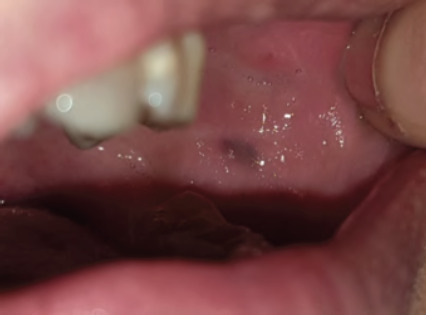
Amalgam tattoo biopsied of left buccal mucosa

Amalgam tattoos are pigmented lesions present in the oral cavity, usually resulting from the displacement of amalgam particles in soft tissues during common dental restorative procedures. This presents as grey—blue or black pigmentation on the oral mucosa. It is usually round and uniformly pigmented and can be present on the buccal mucosa, lips, tongue, floor of the mouth, palate and gingiva (**Figure 28**).^38^

#### Treatment

No treatment is required, but excision using laser may be considered if an amalgam tattoo is present in aesthetic regions of the oral cavity.^38^

### Hairy Tongue

Hairy tongue is a benign and painless condition resulting in the filiform papilla of the tongue being greatly elongated.^39^ It affects both genders but is more prevalent in males. The incidence is approximately 0.5% of the population.^39^

#### Clinical Features

**Figure 29:**
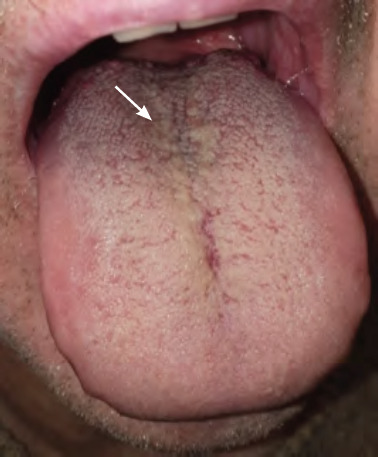
Hairy tongue on dorsal surface of tongue

It is caused by defective desquamation and hypertrophy of the cells in the central column of the filiform papillae, leading to the prevalence of chromogenic bacteria. It can present with significant discolouration: white, yellow, green, brown or black (**Figure 29**). Aetiological factors include poor oral hygiene, antibiotic and psychotropic agents, xerostomia, mouthwashes, and smoking and alcohol use.^39,40^ Discolouration can occur due to tobacco, coffee, tea and food.^39^

#### Treatment

Management of hairy tongue includes minimising aetiological factors such as smoking and coffee. Regular tongue brushing is also recommended, using a tongue scraper or toothbrush. Hairy tongue can be managed effectively with appropriate oral hygiene and patient education. In rare cases, surgical removal of papillae may be required.^40^

## Conclusion

The oral cavity is often an under-examined area in general practice. Increased knowledge about the common presentations of oral lesions can improve practitioner confidence in conducting oral examinations and managing any identified pathology.

Importantly, general practitioners should keep in mind the red flags in oral pathology that may indicate malignancy. A good rule of thumb is to refer any lesion not resolving after 3 weeks for specialist review. Additional red flags include non-healing ulcers, ill-fitting prostheses, erythroplakia, leukoplakia, ulceration with fissures, and lesions with features of induration and fixation.

Particular attention in oral examination should be provided to patients who present with identifiable risk factors, such as smoking, betel nut or areca nut consumption, solar radiation, HPV infection and immunocompromised state.

**How does this paper make a difference to general practice?**

- This is an educational paper, designed to improve the clinical knowledge of general practitioners in oral health.
- This paper categorises common oral lesions by presentation and provides clinical photographs to assist general practitioners in recognising and diagnosing many commonly encountered oral lesions.
- This paper also discusses risk factors and management of common oral lesions and highlights red flags for clinicians to watch out for.
